# The effect of dietary carbohydrate quality on depression and anxiety levels in adolescents

**DOI:** 10.3389/fnut.2025.1689004

**Published:** 2025-11-19

**Authors:** Taha Gökmen Ülger, Bensu Sürücü, Ayşe Ebrar Menekşe, Yağmur Çakmak, Şura Fidan, Berre İncekara

**Affiliations:** 1 Department of Nutrition and Dietetics, Bolu Abant Izzet Baysal University, Bolu, Türkiye; 2 Department of Nutrition and Dietetics, Yeditepe University, İstanbul, Türkiye

**Keywords:** carbohydrate quality, glycemic index, depression, anxiety, adolescent

## Abstract

**Background:**

Given that the quality of carbohydrate intake, rather than quantity alone, may influence psychological health, particularly during adolescence, this study aimed to evaluate the relationship between dietary carbohydrate quality and depression and anxiety levels in adolescents.

**Methods:**

This cross-sectional study was conducted with a sample of 192 second-year high school students from Bolu (Turkey). Participants’ 24-h retrospective food consumption records were collected, and the data were analyzed through the Nutrition Information System to calculate the energy, macro, and micronutrient content of their diets. Dietary carbohydrate quality was evaluated using a quintile-based scoring system created according to criteria such as glycemic index, total fiber intake, the ratio of whole grains to total grains, and the ratio of solid carbohydrates to total carbohydrates, with total scores ranging from 4 to 20 points. Depression and anxiety levels were assessed using the Beck Depression Inventory and the Beck Anxiety Inventory.

**Results:**

The analysis results revealed a statistically significant decrease in depression and anxiety levels as dietary carbohydrate quality increased (*p* < 0.05). The group with the lowest carbohydrate quality had the highest levels of depression and anxiety, and these individuals were found to have higher energy/fiber and carbohydrate/fiber ratios (*p* < 0.001). Moreover, adolescents with a habit of skipping main meals were found to have significantly higher anxiety scores (*p* < 0.05). Sodium, phosphorus, and vitamin A intakes exceeded recommended levels among male adolescents, whereas both sexes demonstrated generally insufficient intake of dietary fiber and various micronutrients, including vitamins E and C, riboflavin, pyridoxine, folate, potassium, calcium, magnesium, iron, and zinc.

**Conclusion:**

The results indicate that adolescents with low carbohydrate quality and irregular meal patterns are at risk of depression and anxiety. Nutritional interventions aimed at addressing imbalances in the intake of macro and micronutrients and improving carbohydrate quality are thought to play an important role not only in preventing metabolic diseases but also in supporting psychological well-being.

## 1 Introduction

Although total carbohydrate intake has not exhibited substantial variation over the past 70 years, it is well recognized that notable differences exist in the types of carbohydrates consumed across various regions of the world. It has been observed that the consumption of low-quality carbohydrate foods has become increasingly prevalent, particularly in economically less developed countries (1). Adolescents represent the population group most affected by this situation. In a study examining how dietary carbohydrate intake and carbohydrate quality changed among adolescents over the 20-year period spanning the late 20th and early 21st centuries, it was found that total carbohydrate and added simple sugar intake increased over time, whereas whole grain and dietary fiber intake declined (2). This situation is considered one of the contributing factors to the recent increase in the prevalence of nutrition-related diseases among adolescents, such as prediabetes, type 2 diabetes, obesity, and metabolic syndrome (3).

Apart from metabolic disorders, a significant increase in the prevalence of depression and anxiety is observed during the transition from childhood to adolescence, and this situation triggers adverse dietary behaviors (4). On the other hand, unhealthy dietary behaviors and an imbalanced diet pattern influence depression and anxiety in adolescents (5). In other words, there exists a bidirectional relationship between dietary patterns and depression and anxiety. Mood disorders characterized by depression and anxiety can influence individuals’ food choices, as well as the consumption of certain foods that may impact symptoms related to these conditions (6, 7). Although several prospective and epidemiological studies have provided strong evidence for the relationship between dietary patterns and depression and anxiety (8, 9), the impact of dietary carbohydrate quality on these mood disorders in adolescents has yet to be fully elucidated. The results of the limited number of studies evaluating the relationship between dietary carbohydrate quality and depression and anxiety, however, are inconsistent (10, 11). Nevertheless, recent advances in nutritional neuroscience have begun to explore the potential physiological mechanisms underlying these associations.

Emerging evidence suggests that the association between dietary carbohydrate quality and mood disorders may be mediated through several interconnected physiological mechanisms. Diets with a high glycemic index and load can cause rapid fluctuations in blood glucose, influencing serotonin and dopamine synthesis through altered tryptophan transport and insulin dynamics (12). Recurrent glycemic variability may activate the hypothalamic–pituitary–adrenal axis and elevate cortisol levels, contributing to anxiety and mood instability (13). Moreover, excessive intake of refined carbohydrates promotes systemic inflammation and oxidative stress, both implicated in the pathophysiology of depression and anxiety (14). Another mechanism involves the gut–brain axis, where fiber-rich and low-glycemic diets enhance microbial diversity and short-chain fatty acid production, exerting anti-inflammatory and neuroprotective effects (15). Conversely, low-quality carbohydrate diets may disrupt gut microbiota balance and increase neuroinflammatory responses (16). These mechanisms collectively highlight the biological pathways through which carbohydrate quality may influence psychological well-being.

The literature indicates that refined foods with high added simple sugar content and low fiber content have a high risk of leading to food addiction, and that the prevalence of food addiction is higher among adolescents compared to other age groups (17, 18). These foods, with a high potential for food addiction, are also characterized by low carbohydrate quality due to their high simple sugar content and low fiber content. It is believed that these types of foods may contribute to the increase in the prevalence of depression and anxiety in this age group by reducing dietary diversity in adolescents’ daily diets and promoting the consumption of foods with low carbohydrate quality.

In conclusion, while carbohydrate quality is recognized as an important risk factor in the etiology of metabolic diseases, it is likely also a significant determinant of psychological well-being. One possible reason for the high prevalence of depression and anxiety in adolescents may be the low dietary carbohydrate quality in this population. This study aims to assess adolescents’ dietary patterns based on the Turkish Nutrition Guide (TNG) reference data (19) and determine the impact of dietary carbohydrate quality on depression and anxiety levels.

## 2 Materials and methods

### 2.1 Study setting, duration, and participants

The study was conducted with adolescent students attending high school in Bolu (Turkey), aged within the adolescent range, and without any chronic illnesses that could affect food choices. To ensure homogeneity within the sample group, data was collected solely from second-year high school students (mean age 16). The sample size was calculated using the G-Power program (version 3.1.9.7), with an effect size of 0.5, a significance level of 0.05, and 90% power, resulting in a minimum of 132 participants. Data were collected through face-to-face surveys between December 15, 2024, and February 15, 2025.

### 2.2 Anthropometric measurements

The students’ body weight, height, waist circumference, and neck circumference measurements were performed by the researchers on the study team in environments designated by the school where the data were collected. Height was measured with the individual barefoot, with feet together and the head in the Frankfurt plane. Body weight was also measured without shoes and in light clothing. For waist circumference measurement, the midpoint between the lowest rib and the iliac crest was identified, and the measurement was taken at this level. Neck circumference was measured with the individual standing upright and the head in a natural position; the measurement was taken just below the thyroid cartilage (Adam’s apple) using a flexible, non-stretchable tape. For body composition analysis, a Tanita BC 545 N digital scale was used. All anthropometric measurements were performed by a single evaluator to ensure consistency and minimize measurement variability. The measurement procedures for height, weight, waist, and neck circumference were conducted in accordance with the standardized anthropometric techniques described by Lohman et al. (20).

### 2.3 Determination of nutrient intake levels and calculation of dietary carbohydrate quality

Dietary intake data were obtained from the adolescents participating in the study using the 24-h recall method, and a total of three 24-h recalls were collected for each participant on non-consecutive days, including two weekdays and one weekend day, to better represent usual dietary intake and account for variations between weekdays and weekends. The mean of the 3 days was calculated to estimate participants’ average daily nutrient intakes. The mean of the 3 days was calculated to estimate participants’ average daily nutrient intakes, and the collected data were evaluated using the Nutrition Information System (BeBİS) program to determine the nutrient intake levels of the adolescents. The dietary carbohydrate quality of the adolescents was determined according to the method of Sawicki et al. (21) (Table 1). In this method, dietary carbohydrate quality is calculated by considering the total fiber intake level, the ratio of whole grains to total grains, glycemic index, solid carbohydrates, and the ratio of total carbohydrates. All parameters to be used in the calculation were considered using BeBİS data, and only the glycemic index assessment was based on the international glycemic index value table by Foster-Powell et al. (22).

**Table 1:** Carbohydrate quality index (CQI) calculation criteria.

CQI component	Score range	Minimum score criterion	Maximum score criterion
Glycemic index	1–5	Highest quintile of glycemic index	Lowest quintile of glycemic index
Dietary fiber intake (g/day)	1–5	Lowest quintile of dietary fiber intake	Highest quintile of dietary fiber intake
Whole grain / total grain ratio	1–5	Lowest quintile of the ratio	Highest quintile of the ratio
Ratio of solid carbohydrates to total (solid + liquid) carbohydrates	1–5	Lowest quintile of the ratio	Highest quintile of the ratio
Total CQI score	4–20		

CQI, Carbohydrate Quality Index.

### 2.4 Determination of depression and anxiety levels

To assess the levels of depression and anxiety, the Beck Depression Inventory (BDI) and the Beck Anxiety Inventory (BAI), developed by Aaron T. Beck, were used. Both scales consist of 21 items, with each item being rated on a scale from 0 to 3, and the total score reflects the level of the respective psychological condition. The Turkish validity and reliability of the BDI were established by Hisli (23), and the BAI by Ulusoy (24). In both the BDI and BAI, higher total scores indicate higher levels or severity of depression and anxiety.

### 2.5 Statistical analysis of the data

IBM SPSS Statistics (Version 22) software was used for data analysis. The normality of continuous variables was assessed using the Kolmogorov–Smirnov test, along with examination of skewness and kurtosis values and visual inspection of histograms and Q–Q plots. Variables showing a normal distribution were analyzed using parametric tests, whereas categorical variables were evaluated using the chi-square test. Descriptive statistics were reported as mean ± standard deviation for numerical variables and as percentages and frequencies for categorical variables. Independent Samples t-test was used to compare the means between two groups, while One-Way ANOVA was applied to determine if there were differences among more than two groups. For variables that showed significant differences in ANOVA, the Tukey post-hoc test was used to identify the differences between groups. Chi-Square test was used to examine the relationship between categorical variables. A significance level of *p* < 0.05 was considered statistically significant.

### 2.6 Ethics

Ethical approval for the study was obtained from the Ethics Committee for Social Science Human Researches at Bolu Abant İzzet Baysal University (Protocol No. 2024/215). After receiving ethical approval, institutional permission was obtained from the Ministry of National Education (Application No: MEB.TT.2024.005721). Permissions for the use of the scales, which ensured their Turkish validity and reliability, were obtained from the authors, and all stages of the study were conducted in accordance with the Helsinki Declaration.

## 3 Results

The study was conducted with a total of 192 adolescents, including 108 females and 84 males, and the characteristics of the adolescents are presented in Table 2. The mean age of female adolescents was 15.52 ± 0.58 years, while the mean age for males was 15.57 ± 0.50 years. The gender-specific difference in the mean age of adolescents was not statistically significant (*p* > 0.05). Regarding the anthropometric findings of the participants, the mean BMI of female adolescents was 22.53 ± 4.43 kg/m^2^, height was 160.63 ± 5.42 cm, body weight was 58.15 ± 11.89 kg, waist circumference was 71.54 ± 8.94 cm, and neck circumference was 31.67 ± 2.26 cm. In male adolescents, the mean BMI was found to be 22.9 ± 4.96 kg/m^2^, height 172.67 ± 7.6 cm, body weight 68.8 ± 17.77 kg, waist circumference 79.34 ± 10.6 cm, and neck circumference 35.55 ± 2.67 cm. While the difference in BMI values between sexes was not statistically significant (*p* > 0.05), male adolescents had significantly higher mean values for height, body weight, waist circumference, and neck circumference (*p* < 0.001). Regarding body fat percentage, the mean value was significantly higher in female adolescents (24.52 ± 8.98) compared to male adolescents (14.63 ± 9.28; *p* < 0.001).

**Table 2:** Dietary pattern data and anthropometric findings for adolescents.

Variable	Women (*n* = 108)		Men (*n* = 84)		*p*
	Mean	SD	Mean	SD	
Age (year)	15.52	0.58	15.57	0.5	>0.05
BMI (kg/m^2^)	22.53	4.43	22.9	4.96	>0.05
Height (cm)	160.63	5.42	172.67	7.6	**<0.001**
Weight (kg)	58.15	11.89	68.8	17.77	**<0.001**
Waist Circumference (cm)	71.54	8.94	79.34	10.6	**<0.001**
Neck Circumference (cm)	31.67	2.26	35.55	2.67	**<0.001**
Body Fat Percentage (%)	24.52	8.98	14.63	9.28	**<0.001**
Anxiety Score	14.62	9.07	10.32	6.66	**<0.001**
Depression Score	12.77	6.83	11.51	6.04	>0.05
CQI	10.35	2.94	10.42	2.57	>0.05
Energy	1,150,46	464,87	1,554,69	766,85	**<0.001**
Protein (g)	41.28	18.01	65.63	43.91	**<0.001**
Protein (Energy %)	15.3	5.01	17.5	5.87	**=0.005**
Protein (g/kg)	0.74	0.37	0.98	0.56	**<0.001**
Lipid (g)	48.02	22.47	66.18	39.94	**<0.001**
Lipid (Energy %)	37.4	10.03	37.4	11.97	>0.05
Carbohydrate (g)	135.68	66.98	171.46	91.16	**=0.002**
Carbohydrate (Energy %)	47.3	11.45	45.1	12.61	>0.05
Fiber (g)	11.10	5.8	13.93	7.77	**=0.004**
PUFA (g)	9.13	6.56	13.42	10.21	**=0.001**
Cholesterol (mg)	196.6	150.8	381.37	388.7	**<0.001**
Number of main meals (n)					**<0.001** ^**#**^
≥3	43		57		
<3	65		27		
Number of snacks	1.39	0.83	1.24	0.92	>0.05

#Chi square test; other analyses were conducted using the independent samples *t*-test. CQI, Carbohydrate Quality Index; BMI, Body Mass Index; PUFA, Polyunsaturated Fatty Acids. Bold values denote statistical significance (*p* < 0.05).

According to the findings on adolescents’ daily energy and macronutrient intake, female adolescents consumed an average of 1150.46 ± 464.87 kcal of energy, 41.28 ± 18.01 g of protein, 48.02 ± 22.47 g of fat, and 135.68 ± 66.98 g of carbohydrates. The average values for male adolescents were determined as 1554.69 ± 766.85 kcal for energy, 65.63 ± 43.91 g for protein, 66.18 ± 39.94 g for fat, and 171.46 ± 91.16 g for carbohydrates. The differences in these values between genders were statistically significant (*p* < 0.05).

In terms of the macronutrient source of daily dietary energy, it was determined that for female adolescents, 15.3 ± 5.01% of the energy came from proteins, 37.4 ± 10.03% from fats, and 47.3 ± 11.45% from carbohydrates. For male adolescents, the percentage of dietary energy from fats was the same as females (37.4 ± 11.97%), while the percentage from proteins and carbohydrates was 17.5 ± 5.87% and 45.1 ± 12.61%, respectively. In the specified ratios, although there were no statistically significant differences in the carbohydrate and fat percentages between genders (*p* > 0.05), the percentage of daily energy derived from protein was significantly higher in male adolescents (*p* = 0.005). Additionally, the protein intake per body weight for male adolescents (0.98 ± 0.56 g) was significantly higher than that of female adolescents (0.74 ± 0.37 g).

In terms of daily intake of fiber, polyunsaturated fatty acids (PUFAs), and cholesterol, male adolescents had significantly higher intake levels compared to females (*p* < 0.05). The average daily fiber intake for males was 13.93 ± 7.77 g, PUFA intake was 11.10 ± 5.8 g, and cholesterol intake was 381.37 ± 388.7 mg. For female adolescents, these values were 11.10 ± 5.8 g, 9.13 ± 6.56 g, and 196.6 ± 150.8 mg, respectively.

The results related to depression and anxiety scores revealed that the anxiety score of female adolescents (14.62 ± 9.07) was significantly higher than that of male adolescents (10.32 ± 6.66; *p* < 0.001). However, no statistically significant difference was observed between genders regarding the depression score (female: 12.77 ± 6.83; male: 11.51 ± 6.04; *p* > 0.05). As for dietary carbohydrate quality, it was found to be 10.35 ± 2.94 in female adolescents and 10.42 ± 2.57 in male adolescents, with no statistically significant gender-specific difference (*p* > 0.05).

The findings regarding main meal consumption indicated that 100 adolescents consumed three or more main meals per day, while 92 consumed fewer than three. The difference in main meal consumption frequency between female and male adolescents was statistically significant (*p* < 0.001). Furthermore, a higher proportion of female adolescents (60.2%) skipped meals compared to their male counterparts (32.1%). However, the average number of snacks consumed by female adolescents (1.39 ± 0.83) was slightly higher than that of male adolescents (1.24 ± 0.92), but this difference was not statistically significant (*p* > 0.05).

**Figure 1:**
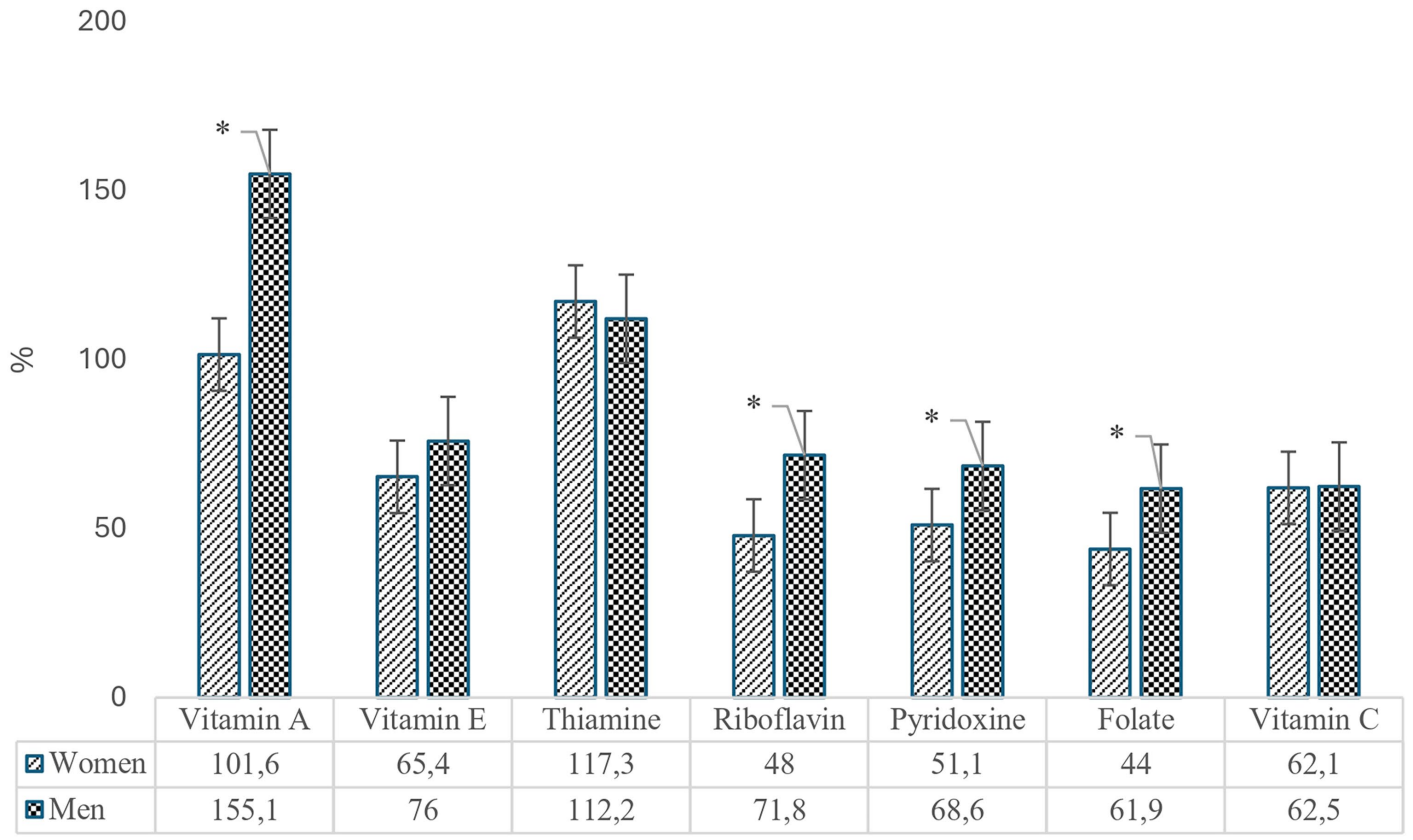
Vitamin intake rates of adolescents according to TNG recommendations (*Indicates statistically significant difference).

The daily vitamin intakes of adolescents were compared with the recommended daily intake levels for vitamins as outlined in the 2022 Turkish Nutrition Guide (TNG), with the corresponding ratios presented in Figure 1. The findings indicated that both female and male adolescents had average intakes of vitamin A and thiamine above the recommended daily levels. The ratio of daily vitamin A intake to the TNG reference value was 155.1% for male adolescents and 101.6% for female adolescents. The gender difference in vitamin A intake was statistically significant (*p* < 0.05). While the average thiamine intake for both genders exceeded the reference level (female: 117%, male: 112%), the gender difference in thiamine intake was not statistically significant (*p* > 0.05).

The average intake levels of riboflavin, pyridoxine, folate, vitamin E, and vitamin C were found to be below the reference values specified in the TNG for both genders. Folate was identified as the vitamin with the lowest intake relative to the reference values among adolescents. Female adolescents consumed 44% of the recommended daily intake of folate, while male adolescents consumed 61.9%. The difference between genders in the percentages of recommended intake fulfillment for folate was statistically significant (*p* < 0.001). Gender-specific differences in the percentage of requirements met for riboflavin and pyridoxine were also statistically significant (*p* < 0.01), with male adolescents having higher intake levels (riboflavin: 71.8%; pyridoxine: 68.6%) compared to female adolescents, whose intake levels were 48% for riboflavin and 51.1% for pyridoxine. For vitamins E and C, the average intake levels as a percentage of the recommended daily intake were 65.4 and 62.1% in female adolescents, respectively, while in male adolescents, these percentages were 76 and 62.5%. No statistically significant gender differences were found for the intake of vitamins E and C (*p* > 0.05).

**Figure 2:**
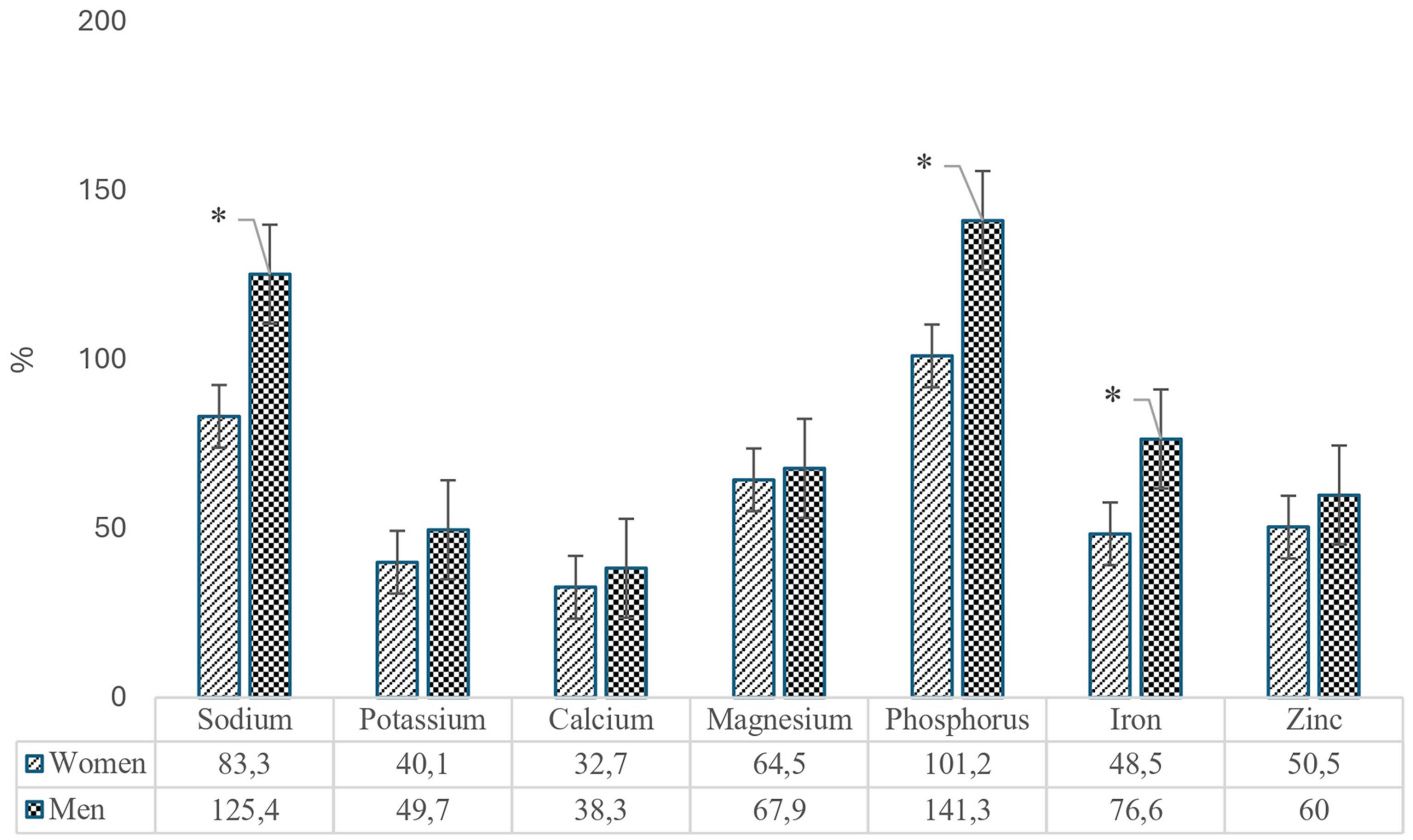
Mineral intake rates of adolescents according to TNG recommendations (*Indicates statistically significant difference).

The daily mineral intakes of adolescents were compared with the recommended daily intake levels for minerals, as specified in the Turkish Nutrition Guide (TNG), and the corresponding ratios are presented in Figure 2. The results indicated that the average daily sodium intake of male adolescents exceeded the recommended level by 25.4%, with a statistically significant gender-specific difference in sodium intake (*p* < 0.001). Both female and male adolescents had average phosphorus intakes exceeding the reference values (female: 101.2%; male: 141.3%), and the gender-based difference was statistically significant (*p* < 0.001). In contrast, calcium was identified as the mineral with the lowest intake relative to the reference values for both genders (female: 32.7%; male: 38.3%). However, the gender-specific difference in calcium intake was not statistically significant (*p* > 0.05).

The intake levels of potassium, iron, and zinc among female adolescents were found to be approximately half or below the reference values. Specifically, the intake levels for female adolescents were 40.1% for potassium, 48.5% for iron, and 50.5% for zinc. In contrast, male adolescents exhibited intake levels of 49.7% for potassium, 76.6% for iron, and 60% for zinc. The intake of magnesium was similar across genders, with female adolescents at 64.5% and male adolescents at 67.9%. Of these minerals, only iron intake demonstrated a statistically significant gender difference (*p* < 0.001), while the differences in magnesium, potassium, and zinc intake were not statistically significant (*p* > 0.05).

The quintiles based on adolescents’ dietary carbohydrate quality, along with corresponding data on general depression and anxiety scores, energy-to-fiber ratio (kcal/g), carbohydrate-to-fiber ratio (g/g), and anthropometric measurements, are presented in Table 3. The findings revealed that the group with the lowest carbohydrate quality exhibited the highest anxiety (15.08 ± 7.37) and depression (13.98 ± 5.24) scores. Conversely, the lowest anxiety score was observed in the Q4 quintile (10.21 ± 7.94), while the lowest depression score was found in the Q5 quintile (9.8 ± 4.42). Statistically significant differences were noted between the quintiles for both anxiety and depression scores (*p* = 0.033 and *p* = 0.039), with a near-linear decrease in both parameters as dietary carbohydrate quality improved.

**Table 3:** Depression and anxiety scores, dietary fiber patterns, and anthropometric findings according to dietary carbohydrate quality.

Variable	CQI										*p*
	Q1		Q2		Q3		Q4		Q5		
	Mean	SD	Mean	SD	Mean	SD	Mean	SD	Mean	SD	
Anxiety score	15.08	7.37	13.93	9.45	11.48	8.32	10.21	7.94	10.48	6.92	**0.033**
Depression score	13.98	5.24	13.09	6.29	11.33	8.42	10.85	7.23	9.8	4.42	**0.039**
Energy-to-fiber ratio (kcal/g)	156.1	85.5	119.7	56.6	131.9	64.9	103.2	42.2	102.6	61.3	**0.001**
Carbohydrate-to-fiber ratio (g/g)	17.32	10.8	12.92	4.86	14.06	5.3	11.4	4.6	10.15	3.54	**<0.001**
Body fat percentage (%)	20.05	10.55	21.22	10.83	20.82	10.7	17.1	9.01	21.56	9.87	0.39
Waist circumference (cm)	74.95	10.97	74.98	12.16	76.23	10.96	74.54	7.63	73.96	8.2	0.949
BMI (kg/m^2^)	22.93	5.43	22.76	4.85	22.98	4.86	22.16	3.02	22.48	4.45	0.947
Height (cm)	165.2	8.3	164.8	9.27	167.4	9.7	167.6	8.8	165.4	7.5	0.522
Weight (kg)	63	17.2	62.6	18.6	64.36	14.38	62.48	11.3	61.3	11.8	0.970

BMI, Body Mass Index, All analyses were performed using one-way ANOVA. Bold values denote statistical significance (*p* < 0.05).

The findings regarding the energy-to-fiber and carbohydrate-to-fiber ratios, which are commonly used methods for evaluating dietary carbohydrate quality in the literature, revealed that as dietary carbohydrate quality increased, both ratios decreased. The highest energy-to-fiber ratio was observed in the Q1 quintile, while the lowest ratio was found in the Q5 quintile. The difference in energy-to-fiber ratios between the quintiles was statistically significant (*p* = 0.001). Similarly, the highest carbohydrate-to-fiber ratio was found in the Q1 quintile, and the lowest in the Q5 quintile, with a statistically significant difference between the quintiles (*p* < 0.001).

The findings related to dietary carbohydrate quality and anthropometric parameters (body fat percentage, waist circumference, BMI, height, and body weight) revealed no statistically significant differences between the quintiles for any of the anthropometric measures (*p* > 0.05).

**Figure 3:**
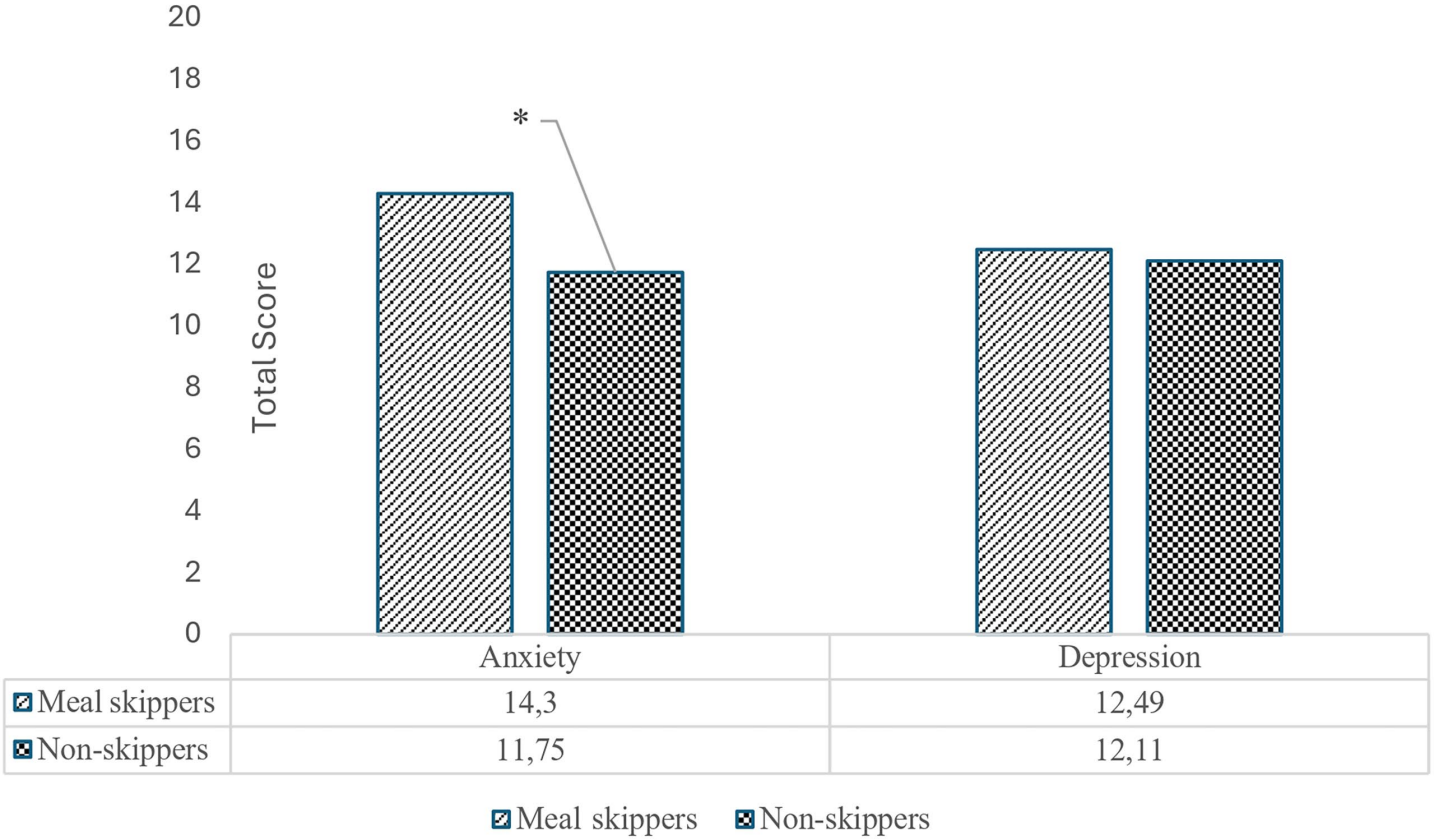
Anxiety and depression levels of adolescents with and without the habit of skipping main meals.

The findings suggest that skipping main meals may be a significant predictor of anxiety scores in adolescents (Figure 3). Adolescents who regularly skipped main meals (n = 92) had higher anxiety scores (14.3 ± 8.9), which were significantly higher compared to those who did not skip meals (11.75 ± 7.64; *p* = 0.039). However, no statistically significant difference was observed in depression scores between adolescents with (12.49 ± 6.31) and without (12.11 ± 6.70) the habit of skipping main meals (*p* > 0.05).

## 4 Discussion

### 4.1 Main findings of this study

Findings from the present study indicate that, the average BMI, body weight, and height values of the adolescents were within the age- and sex-specific reference ranges established for the Turkish population (25). The proportion of dietary energy derived from fat exceeded the reference values set by the TNG (19), representing a potential risk factor with regard to healthy eating principles for children and adolescents. Psychological assessment results indicated that participants’ mean depression and anxiety scores were at a mild level. Furthermore, the intake of key micronutrients including riboflavin, pyridoxine, folate, vitamins E and C, calcium, iron, potassium, zinc, and magnesium was found to be inadequate. Conversely, intakes of sodium, phosphorus, vitamin A, and thiamine among male adolescents exceeded recommended levels. Notably, a decline in dietary carbohydrate quality was associated with higher depression and anxiety scores, underscoring the potential influence of carbohydrate quality on mental health.

### 4.2 Anthropometric and energy intake characteristics, underreporting tendencies

Based on the dietary intake records of the participants, the average energy intake was found to be significantly lower than the energy requirements established by the TNG, which are based on physical activity levels (sedentary, moderately active, active, very active). This discrepancy is believed to stem from common behavioral tendencies in adolescents, such as underreporting or concealing actual food intake. Such tendencies are especially pronounced in those with prevalent fast-food dietary habits or who eat in unstructured environments during social gatherings (26). When self-reporting methods (e.g., food diaries or 24-h dietary recalls) are employed, adolescents often struggle to accurately estimate portion sizes or fail to report foods consumed outside the home, which may compromise the accuracy of the data (27). Furthermore, mood and social acceptance pressures particularly among female adolescents who experience societal pressure regarding body image and diet compliance can lead to the concealment of unhealthy food choices (28, 29).

The literature indicates that food intake is systematically underreported among adolescents, with estimates suggesting that energy intake may be underreported by as much as 41% (30). The use of various dietary assessment methods, such as food frequency questionnaires, tends to result in higher reported food intake compared to food diaries or retrospective recalls. This suggests that individuals are more likely to underreport their intake when shorter-term assessment methods are employed (31). Repeated evidence of underreporting, combined with adolescents’ limited knowledge about food content and preparation methods, further compromises dietary data accuracy (26, 32). Consequently, these findings raise concerns about the reliability of diet data obtained through self-reporting in adolescents. Therefore, employing multifaceted dietary assessment approaches that consider adolescents’ developmental and psychological characteristics is essential to improve reporting accuracy and willingness to disclose actual consumption (33, 34).

No evidence of chronic energy deficiency or insufficiency was found in the anthropometric measurements of height and body weight among the adolescents. The average height values for both female and male adolescents were found to be very close to the age-specific median values established in the TNG (for females, 15 years: 162 cm, 16 years: 163 cm; for males, 15 years: 169 cm, 16 years: 173 cm). In contrast, the average body weight values were >10% higher than the corresponding age group median values (for females, 15 years: 52.8 kg, 16 years: 54.7 kg; for males, 15 years: 56.6 kg, 16 years: 61.3 kg). Taken together, the consistency between dietary intake records and anthropometric measurements highlights the importance of using multiple complementary methods to accurately assess adolescents’ food consumption. This issue also has implications for interpreting nutrient adequacy and psychological associations discussed in the following sections.

### 4.3 Macronutrient intake and dietary patterns in adolescents

Studies evaluating adolescents’ daily dietary patterns indicate that these patterns are often inadequate both quantitatively and qualitatively, which may negatively impact long-term health outcomes (35, 36). Additionally, the distribution and sources of macronutrients in the daily diet are a cause for concern. Adolescents typically consume an imbalanced diet high in sugar and fat, which may lead to long-term health issues such as obesity and cardiovascular diseases. For example, saturated fats contribute approximately 11.6% of the energy intake in adolescents aged 12–15 years, while monounsaturated and polyunsaturated fats account for 12.7 and 6.8%, respectively (37). For instance, during the COVID-19 pandemic, several studies have reported that foods high in energy and saturated fats were frequently consumed, whereas fruit and vegetable intake showed only limited improvement, indicating a deterioration in overall dietary quality (38). The findings of this study also revealed a high proportion of energy derived from fat in adolescents’ daily diets, with notably elevated daily cholesterol intake observed among male adolescents.

The findings indicate that adolescents’ protein intake is within the normal range; however, the literature highlights that the insufficient consumption of high-quality protein sources, such as lean meats, legumes, and dairy products, often results in protein quality being below optimal levels (39, 40). While many adolescents meet the minimum protein requirements through their diet, emerging evidence suggests that the protein consumed primarily originates from sources with significantly lower nutritional value (39, 40). For instance, high-quality protein sources like lean meats, legumes, and low-fat dairy products provide a well-balanced profile of essential amino acids with minimal unwanted fat content. In contrast, many adolescent diets rely on protein sources high in fat, cholesterol, or added sugars, which could contribute to adverse health outcomes over time (41). Furthermore, excessive intake of certain types of animal protein has been linked to abdominal obesity and other metabolic disorders. Studies have established a relationship between animal protein consumption and overall body fat accumulation in adolescents (42). This mismatch between the quantity and quality of protein intake suggests that, even if adolescents adhere to the recommended protein intake guidelines, they may still be at risk of inadequate nutrition in terms of optimal growth, lean body mass development, and long-term metabolic health.

### 4.4 Micronutrient deficiencies and excesses

The present study found that adolescents’ intake of essential micronutrients, including riboflavin, pyridoxine, folate, vitamins E and C, as well as calcium, iron, potassium, zinc, and magnesium, was below the recommended levels. In contrast, particularly among male adolescents, the intake of sodium, phosphorus, vitamin A, and thiamine exceeded the reference values. These findings are consistent with the reported micronutrient deficiencies and imbalances documented in the literature. Studies on adolescent micronutrient intake indicate that deficiencies in folate and B-complex vitamins, particularly riboflavin and pyridoxine, are common (43). Ayal et al. (44) found that general micronutrient deficiencies are widespread among adolescents and that current dietary habits increase the risk of such deficiencies. Despite the importance of riboflavin and pyridoxine in various metabolic processes, many adolescent groups consume these nutrients in insufficient amounts. Kawade (45) emphasized that the average intake levels of several micronutrients, including zinc, are inadequate among adolescents, and noted that micronutrient deficiencies are prevalent across different geographic regions. Additionally, multiple studies have reported insufficient intake of calcium, zinc, and vitamins E and C among adolescents, and these findings align with those obtained in this study (46, 47).

On the other hand, studies have shown that adolescents living in urban areas often consume excessive amounts of processed, refined foods, fast food, and sugary beverages, resulting in sodium and phosphorus intakes that exceed the recommended levels. These findings are consistent with the existing literature (48, 49). While the literature also notes that some adolescents consume certain micronutrients, such as vitamin A and thiamine, at levels above the recommended intake, these findings have not yet been widely generalized (50).

In summary, the pattern of excessive consumption of processed foods and fast food, combined with a low intake of vegetables, fruits, and dairy products, as reflected in the data, may increase adolescents’ vulnerability to future health issues, including cardiovascular diseases, diabetes, and cancer.

### 4.5 Dietary habits and mental health outcomes

Adolescence is a developmental phase marked by heightened susceptibility to mental health disorders such as depression, stress, and anxiety, which arise from the complex interplay of biological, psychological, and social changes. During this critical period, meal patterns play a significant role in regulating psychological and mental well-being.

The practice of skipping main meals, particularly breakfast and lunch, during adolescence has been significantly associated with mental health problems such as depression and anxiety. The literature emphasizes that regular meal consumption, especially family meals, serves as a protective factor against depressive symptoms in adolescents (51, 52). Additionally, a structured meal routine has been shown to support coping mechanisms for stress, particularly for adolescents facing academic and social pressures (53, 54).

When evaluated at the metabolic level, skipping meals can lead to fluctuations in blood glucose levels, which may contribute to the onset of mood disorders (55). Such fluctuations in glucose homeostasis are known to influence the secretion of neuroendocrine regulators, including insulin and cortisol, thereby exacerbating emotional instability. This disruption in metabolic balance can facilitate the development of unhealthy eating habits, often accompanied by symptoms such as fatigue, irritability, and difficulty concentrating (56). Conversely, existing research indicates that current levels of depression and anxiety negatively influence eating behaviors, leading to an increase in irregular eating patterns, such as meal skipping, thus creating a bidirectional relationship between mood and dietary behavior (55). The findings of this study align with the literature, revealing that adolescents who skipped main meals exhibited significantly higher levels of anxiety.

### 4.6 Carbohydrate quality and mental health

An important determinant of adolescent health is the composition of carbohydrates in their diet. Carbohydrate quality refers to the nutritional and physiological value of carbohydrate-containing foods, influenced by factors such as fiber content, degree of processing, and glycemic response. Literature suggests that, rather than the quantity of carbohydrate intake, its quality plays a crucial role in regulating mood and emotional well-being (57). In academic studies, the quality of dietary carbohydrates is typically assessed using factors such as glycemic index, glycemic load, fiber content, and degree of processing. High glycemic index foods have been identified as risk factors in the pathophysiology of mood disorders, due to their potential to induce glycemic variability, systemic inflammation, and neurochemical imbalances. In contrast, low glycemic index foods have been associated with more favorable neuropsychological outcomes (58). These findings highlight that carbohydrate quality not only influences metabolic balance but may also play a pivotal role in maintaining emotional stability during adolescence. The findings of this study further support this notion, as an increase in the quality of dietary carbohydrates was associated with lower depression and anxiety scores.

A growing body of observational and interventional research has begun to clarify the potential mechanisms linking high-glycemic-index diets to adverse mental health outcomes. In this context, clinical and case-based evidence further supports the association between glycemic regulation and mood symptoms. For example, a case report indicated that reducing the intake of refined carbohydrates was associated with improvements in symptoms of generalized anxiety disorder and hypoglycemia (59). Moreover, a study involving adolescents with type 1 diabetes found a relationship between poor glycemic control and elevated psychological distress (60). Although this association is primarily related to diabetes management, it suggests that glycemic variability may have broader implications for psychological well-being in both diabetic and non-diabetic populations.

The literature has also proposed a mechanistic explanation for the relationship between glycemic index and psychological well-being. Meals composed of high-glycemic-index foods can lead to marked postprandial elevations in blood glucose levels, which may subsequently be followed by reactive hypoglycemia. Such glycemic fluctuations are thought to influence neuroendocrine regulators including insulin and cortisol that play key roles in modulating mood and stress responses. Research on glycemic variability has identified a correlation between acute spikes in blood glucose and heightened anxiety-related traits, suggesting that blood glucose stability may be linked to improved psychological well-being (61). Moreover, a systematic review by Rahimlou et al. (61) found that diets characterized by a low glycemic index may reduce the risk of depression, reinforcing the notion that glycemic stability represents a potentially modifiable factor in mood regulation. While these findings highlight the biological mechanisms linking diet and mental health, it should be acknowledged that several behavioral and contextual factors may also play an important role.

In addition to dietary factors, several external variables may influence both eating behavior and psychological outcomes in adolescents. Physical activity level plays a critical role in regulating energy balance, neuroendocrine function and mood, with lower activity frequently associated with higher depressive and anxiety symptoms (62, 63). Irregular or insufficient sleep can disrupt circadian rhythms and neurotransmitter homeostasis, thereby contributing to emotional dysregulation and heightened anxiety (64). Socioeconomic status affects access to nutrient-dense foods and exposure to psychosocial stressors, both of which may confound the relationship between carbohydrate quality and mental health (65). Cultural and familial dietary practices also shape food preferences and meal patterns and may therefore modify the associations observed across different populations (66). Although these factors were not measured in the present study, acknowledging their potential influence underscores the need to include such variables in future prospective and interventional research to disentangle causal pathways.

Dietary fiber content is a key determinant of carbohydrate quality. A growing body of research indicates that higher fiber intake is inversely associated with symptoms of depression and anxiety. For instance, a systematic review by Aslam et al. (67) demonstrated that increased fiber consumption is linked to a lower risk of these mental health disorders. Similarly, Oddy et al. (68) highlighted that high-fiber, nutrient-dense diets may enhance mental health by mitigating systemic inflammation. Conversely, evidence also suggests a U-shaped relationship between fiber intake and mental health, indicating that both insufficient and excessive fiber consumption may have detrimental effects (69). Mao et al. (70) further emphasized that the protective effects of fiber against depression may differ across demographic groups, and that both the type and source of fiber play a critical role.

In line with this literature, the findings of the present study revealed that as the dietary carbohydrate quality index increased, the carbohydrate-to-fiber and energy-to-fiber ratios declined, while depression and anxiety scores significantly decreased. These results support the notion that adequate fiber intake may serve as a protective factor against the development of depression and anxiety, particularly during adolescence.

Overall, the results of this study are consistent with the growing evidence linking carbohydrate quality to psychological well-being. Dietary interventions aimed at reducing the glycemic index or ensuring adequate fiber intake in adolescents have demonstrated potential for alleviating anxiety symptoms, suggesting that lifestyle changes may serve as complementary tools in the management of mental health. Although no studies in the literature specifically assess the relationship between dietary carbohydrate quality and depression or anxiety in adolescents, the concept of carbohydrate quality is increasingly recognized as a key determinant of health. It is proposed as a more comprehensive measure that extends beyond glycemic index and fiber content alone (71–73). The carbohydrate quality index considers the differential physiological effects of solid and liquid carbohydrates, underscoring the advantages of a holistic approach rather than reliance on isolated metrics such as glycemic index or fiber content. This multidimensional approach provides a broader understanding of the health impact of carbohydrate consumption, offering a more precise method for predicting health outcomes. In line with recent developments in this area, incorporating carbohydrate quality as a central element in dietary guidelines could play a significant role in reducing the risk of chronic diseases and improving overall health outcomes.

### 4.7 Strengths, limitations, and future directions

This study has several notable strengths. It specifically targets an adolescent population, a developmental period in which dietary behaviors and emotional regulation undergo significant change. The comprehensive evaluation of dietary carbohydrate quality through multiple indicators, together with validated measures of depression and anxiety, provides valuable insight into the relationship between diet and psychological health. This multifactorial assessment strengthens the study’s contribution to the growing field of nutritional psychiatry, particularly within adolescent populations. Furthermore, the use of multiple 24-h dietary recalls that included both weekdays and weekends strengthened the representativeness of usual intake and enhanced the reliability of dietary assessment.

However, certain limitations should be acknowledged. The cross-sectional design precludes any inference of causality between dietary carbohydrate quality and mental health outcomes. The use of self-reported dietary data and psychological assessments introduces the possibility of recall and reporting bias. In addition, some potentially influential variables such as physical activity, sleep quality, socioeconomic status, and cultural eating habits were not assessed in detail. These unmeasured factors could partially account for the associations observed in the present study.

Future research should build upon these findings through well-designed longitudinal and interventional studies to elucidate causal mechanisms underlying the relationship between carbohydrate quality and mental health in adolescents. Experimental designs incorporating controlled dietary interventions could help clarify the directionality and magnitude of these effects. Additionally, integrating objective measures such as accelerometry for physical activity, actigraphy for sleep patterns, and biomarkers for dietary intake would improve the accuracy of data collection. Cross-cultural comparisons may also shed light on how sociocultural dietary patterns interact with mental health outcomes. Such approaches will contribute to a more comprehensive understanding of how carbohydrate quality can be leveraged in nutritional strategies aimed at promoting adolescent psychological well-being. Ultimately, integrating these findings into public health and educational strategies could help shape healthier dietary behaviors and improve mental well-being during adolescence.

## 5 Conclusion

The findings of this study suggest that low carbohydrate quality in adolescents’ diets may be associated with increased levels of depression and anxiety. Dietary patterns dominated by refined carbohydrates characterized by a high glycemic index and low fiber content appear to have a detrimental impact on mental well-being. Additionally, the results indicate widespread inadequacies in micronutrient intake among adolescents, particularly in essential nutrients such as folate, riboflavin, and vitamins C and E. These findings underscore the critical importance of enhancing overall dietary quality, not only to support metabolic health but also to promote psychological well-being. Nutritional interventions focused on improving carbohydrate quality may offer a promising approach to supporting adolescent mental health. Accordingly, incorporating carbohydrate quality as a key component in national nutrition strategies targeting this age group is warranted.

## Data Availability

The raw data supporting the conclusions of this article will be made available by the authors, without undue reservation.
